# Edaravone combined with Shuxuening versus edaravone alone in the treatment of acute cerebral infarction: A systematic review and meta-analysis

**DOI:** 10.1097/MD.0000000000032929

**Published:** 2023-03-03

**Authors:** Liang-Da Li, Yue Zhou, Shan-Fen Shi

**Affiliations:** a Department of Neurology, The People’s Hospital Affiliated to Ningbo University, Ningbo, Zhejiang Province, China; b Department of Rheumatology, The People’s Hospital Affiliated to Ningbo University, Ningbo, Zhejiang Province, China.

**Keywords:** acute cerebral infarction, edaravone, meta-analysis, neurologic impairment, Shuxuening

## Abstract

Shuxuening injection (SXN) is a traditional Chinese medicine used in the treatment of cardiovascular diseases. Whether it can provide better outcomes when combined with edaravone injection (ERI) for the treatment of acute cerebral infarction is not well determined. Therefore, we evaluated the efficacy of ERI combined with SXN versus that of ERI alone in patients with acute cerebral infarction.

**Methods:**

PubMed, Embase, Cochrane Library, China National Knowledge Infrastructure, and Wanfang electronic databases were searched up to July 2022. Randomized controlled trials comparing the outcomes of efficacy rate, neurologic impairment, inflammatory factors, and hemorheology were included. Odds ratio or standard mean difference (SMD) with corresponding 95% confidence intervals (CIs) were used to present the overall estimates. The quality of the included trials was evaluated by the Cochrane risk of bias tool. The study was performed according to the Preferred Reporting Items for Systematic reviews and Meta-Analyses.

**Results:**

Seventeen randomized controlled trials were included consisting of 1607 patients. Compared to ERI alone, treatment with ERI plus SXN had a greater effective rate than ER alone (odds ratio = 3.94; 95% CI: 2.85, 5.44; *I*^2^ = 0%, *P* < .00001), a lower National Institute of Health Stroke Scale (SMD= −1.39; 95% CI: −1.73, −1.05; *I*^2^ = 71%, *P* < .00001), lower neural function defect score (SMD= −0.75; 95% CI: −1.06,−0.43; *I*^2^ = 67%, *P* < .00001), and lower level of neuron-specific enolase (SMD= −2.10; 95% CI: −2.85, −1.35; *I*^2^ = 85%, *P* < .00001). ERI plus SXN treatment provided significant improvements in whole blood high shear viscosity (SMD = −0.87; 95% CI: −1.17, −0.57; *I*^2^ = 0%, *P* < .00001), and whole blood low shear viscosity (SMD = −1.50; 95% CI: −1.65, −1.36; *I*^2^ = 0%, *P* < .00001) compared to ERI alone.

**Conclusion:**

ERI plus SXN showed better efficacy than ERI alone for patients with acute cerebral infarction. Our study provides evidence supporting the application of ERI plus SXN for acute cerebral infarction.

## 1. Introduction

Acute cerebral infarction, known as ischemic stroke, is one of the most common cerebrovascular diseases worldwide.^[1]^ It is mainly caused by atherosclerosis and thrombosis in the blood vessels supplying the brain, resulting in stenosis and blockage of the vessels.^[2]^ With a period of blood supply disorder, oxidative stress and neuroinflammation occur, and these will lead to damage to the neural function and necrosis of brain tissue.^[1,3,4]^ Due to the rapid onset and complex clinical symptoms of this disease, it can cause several conditions, such as hemiplegia, sensory dysfunction, and even brain herniation.^[5–9]^ Researchers have proposed that the early rescue of ischemic nerve cells is important for the treatment and prognosis of patients with cerebral infarction.^[10–12]^ With the advancement of medical technology, early thrombolysis, and thrombectomy are important treatments for cerebral infarction.^[13]^ Based on these treatment strategies, how to further improve the prognosis of these patients becomes increasingly apparent.

Edaravone injection (ERI) is a new type of hydroxyl radical scavenger.^[14,15]^ Studies have shown that edaravone can reduce the damage caused by cerebral ischemia-reperfusion,^[16]^ and can reduce cerebral edema.^[17]^ At the same time, this drug has a protective effect on the nerve cells in the ischemic area of the brain.^[18]^ Edaravone can inhibit the degree of nerve cell necrosis and reduce the area of necrosis, to achieve the effect of treating cerebral infarction.^[18,19]^ Clinical studies have shown that edaravone can improve the neurological function of patients with cerebral infarction, and reduce the levels of tumor necrosis factor (TNF) and interleukin (IL)-8, thereby increasing the cure rate and improving the quality of life.^[14]^

Shuxuening injection (SXN) is a Chinese medicine extracted from Ginkgo biloba leaves.^[20,21]^ Ginkgo biloba extract mainly includes ginkgolide and flavonoid glycosides.^[21–23]^ Ginkgolide has a strong antioxidant effect, which can inhibit lipid peroxidation, scavenge oxygen free radicals, and effectively protect injured tissue.^[24,25]^ Studies have shown that this drug has a protective effect on nerve cells in the brain and can effectively relieve the clinical symptoms of patients with cerebral infarction.^[20,26,27]^ However, strong evidence on the effect of SXN combined with ERI injection for acute cerebral infarction is limited.

In recent years, more and more clinical studies have analyzed the efficacy of ERI combined with SXN in the treatment of acute cerebral infarction, but clinical studies are mostly small-sample, single-center randomized controlled trials (RCTs). In addition, the potential underlying mechanism of this combination still needs to be clarified. Therefore, in this study, we systematically evaluated the effects of SXI combined with ERI on the clinical efficacy of improving neural function injury, along with inflammatory factor expression and hemorheology in acute cerebral infarction.

## 2. Methods

This study was not registered. It was performed following the guideline of the Preferred Reporting Items for Systematic Reviews and Meta-Analyses checklist. This is a systematic review and ethics approval is not applicable.

### 2.1. Literature search

A comprehensive literature search was conducted on electronic databases such as PubMed, Embase, Cochrane Library, China National Knowledge Infrastructure, and Wanfang. The retrieval time is from the establishment of the database to July 1, 2022. The main search terms were: Shuxuening injection, Edaravone injection, and acute cerebral infarction. Additionally, references from selected articles and reviews were manually searched for all potentially relevant studies. No language limitation was applied during the search process.

### 2.2. Inclusion and exclusion criteria

#### Inclusion criteria:

Patients: acute cerebral infarction was diagnosed according to adequate diagnostic criteria, cranial computed tomography (CT), and magnetic resonance imaging techniques; Intervention measures: patients were randomly divided into 2 or more groups, and there should be 1 intervention group received ERI combined with SXN, and 1 control group received placebo or ERI; Comparison: there were comparisons based on the grouping methods; Outcomes: outcomes such as efficacy, improvement of neurological function, neurological impairment score, changes in the expression levels of inflammatory factors, changes in hemorheology, and adverse reactions were reported in the included RCTs.

#### Exclusion criteria:

non-original studies (such as meta-analysis, reviews), conference abstracts, case reports, repeated studies, studies that could not extract outcome indicators, studies that did not use SXN combined with ERI in the treatment of acute cerebral infarction, studies without sufficient data, and the full-text of the included studies could not be obtained.

### 2.3. Data extraction and quality assessment

Data extraction and evaluation were done by 2 researchers, independently. And disagreements were resolved by discussion with a third researcher. Extracted data included title, author, publication year, study type, treatment strategy, number of patients in intervention and control groups, efficacy, National Institute of Health Stroke Scale (NIHSS), neural function defect score (NDS), neuron-specific enolase (NSE), IL-6, S100-beta, activities of daily living (ADL), vascular endothelial growth factor (VEGF), TNF-α, C-reactive protein (CRP), IL-6, whole blood high shear viscosity, whole blood low shear viscosity, and safety.

The quality of the included RCTs was assessed using the quality assessment tool provided in Cochrane handbook 5.1.^[28]^ The assessment aspects included methods of randomization, allocation concealment, blind implementation, complete reporting of outcome data, selective reporting, and other sources of bias.

### 2.4. Statistical analysis

RevMan5.4 and STATA16.0 software were used for statistical analysis. The methods are as described previously.^[29]^ Briefly, the odds ratio and 95% confidence interval (CI) were used to evaluate count data, and the mean difference or standard mean difference (SMD) and its standard deviation were used to evaluate quantitative data. For the analysis of heterogeneity among the included studies, the chi-square test and *I*^2^ were used. Heterogeneity was considered to be significant if the chi-square test indicated *P* < .1 or *I*^2^ > 50%. If heterogeneity was observed, a random effects model was used to check the effect of heterogeneity on the combined results, whereas a fixed effects model was used. Funnel plot, Egger, and Begg tests were used to assess publication bias. *P* < .05 indicates that the difference is statistically significant.

## 3. Results

### 3.1. Results of literature search and screening

**Figure 1.**
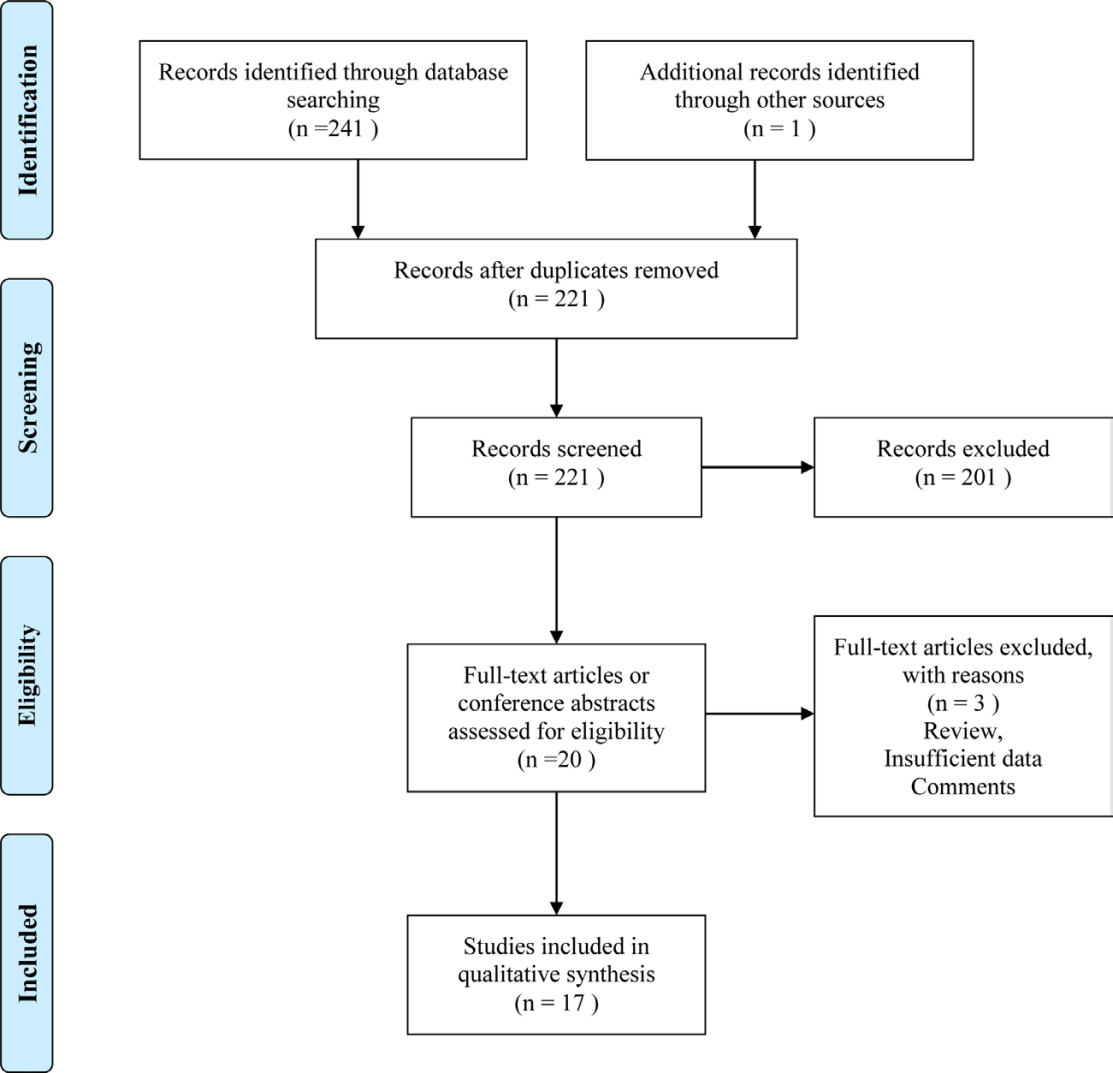
Flow diagram of literature retrieval and screening.

Two hundred forty-two records were retrieved from PubMed, Embase and Cochrane Library, China National Knowledge Infrastructure, and Wanfang database. Twenty-one duplicate studies were excluded. After screening titles and abstracts, 201 studies were excluded. By reading the full text of the remaining articles, a total of 17 RCTs^[30–46]^ involving 1607 patients were included (Fig. 1).

### 3.2. Basic information and quality assessment of the included studies

The main baseline characteristics of the included RCTs are shown in Table 1. Fifteen studies reported clinical efficacy, 7 articles observed changes in NIHSS before and after treatment, 3 articles detected the level of NSE, and 5 articles examined the level of NSE. The study observed changes in the score of neurological deficit, and observed changes in S100-beta, ADL score, IL-6 level, VEGF, whole blood high shear viscosity, and whole blood low shear viscosity. Three articles reported the occurrence of adverse reactions.

**Table 1:** Baseline characteristics of included studies.

Author	Year	Number		Interventions		Endpoints												
		Experimental group	Control group	Experimental group	Control group	Effectiveness	NIHSS	NSE	CRP	NDS	S100-beta	ADL	IL-6	VEGF	Whole blood high shear viscosity	Whole blood low shear viscosity	Ang	TNF-α
Xianda, Wang	2022	40	40	SXN plus ERI	ERI		Yes	Yes									Yes	
Jingjing, Feng	2022	55	55	SXN plus ERI	ERI		Yes	Yes			Yes				Yes	Yes		
Bo, Shi.	2021	31	31	SXN plus ERI	ERI	Yes												
Yuhua, Xu.	2019	56	56	SXN plus ERI	ERI	Yes	Yes	Yes						Yes				
Li, Yang.	2018	39	38	SXN plus ERI	ERI	Yes						Yes			Yes	Yes		
Xianhe, Ye.	2017	24	24	SXN plus ERI	ERI	Yes	Yes											
Zejun, Chen.	2016	49	49	SXN plus ERI	ERI	Yes							Yes					Yes
Pengfei, Wang.	2016	33	34	SXN plus ERI	ERI	Yes					Yes		Yes				Yes	
Jun, Chen.	2015	30	30	SXN plus ERI	ERI	Yes	Yes											
Lin, Li.	2013	82	82	SXN plus ERI	ERI	Yes	Yes					Yes						
Honglian, Wang.	2013	40	40	SXN plus ERI	ERI	Yes	Yes							Yes				
Weihua, Wei.	2012	58	58	SXN plus ERI	ERI	Yes				Yes								
Wei, Zhang.	2012	60	60	SXN plus ERI	ERI	Yes				Yes								
Jiujin, Wei.	2012	35	35	SXN plus ERI	ERI	Yes				Yes								
Jianhong, Chen.	2011	58	50	SXN plus ERI	ERI	Yes												
Haizhu, Ge.	2010	36	34	SXN plus ERI	ERI	Yes				Yes								
Baolin, Wang.	2010	82	83	SXN plus ERI	ERI	Yes			Yes	Yes								

ADL = activies of daily living, CRP = C-reactive protein, ERI = edaravone injection, IL-6 = interleukin-6, NDS = neural function defect score, NIHSS = National Institute of Health Stroke Scale, NSE = neuron specific enolase, SXN = Shuxuening injection. TNF-α = tumor necrosis factor-α, VEGF = vascular endothelial growth factor.

**Figure 2.**
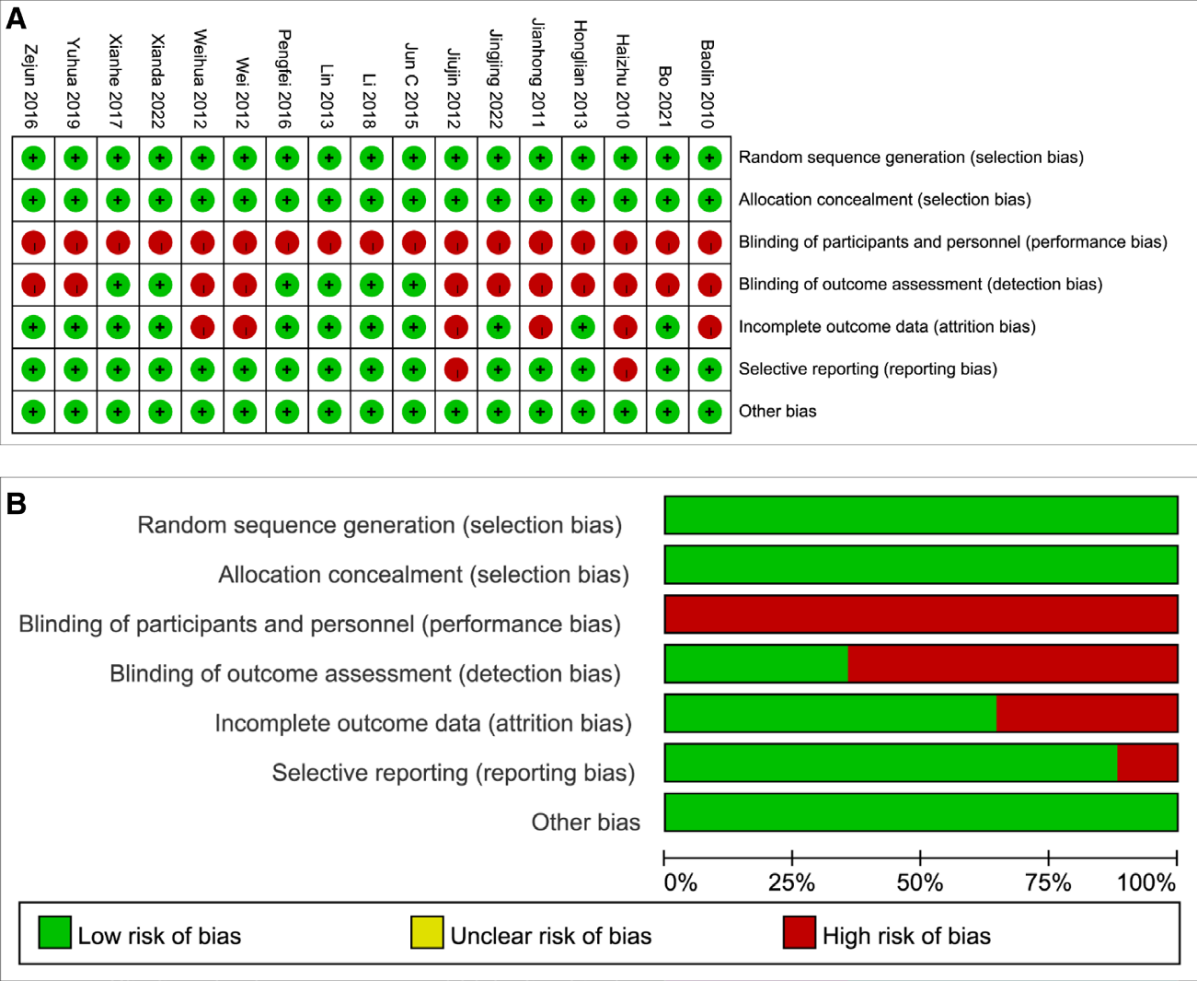
Quality assessments of the included studies. (A) Risk of bias summary. (B) Risk of bias graph.

The quality of included studies was assessed using the tool provided in the RevMan 5.4 software. Among them, 10 studies were of high quality, and the remaining studies were rated as moderate quality. None of the included studies mentioned allocation concealment or blinding, and a high risk of selection bias may exist among the outcome indicators. The specific assessment information on the risk of bias is shown in Figure 2.

### 3.3. Meta-analysis results

#### 3.3.1. Curative effectiveness.

**Figure 3.**
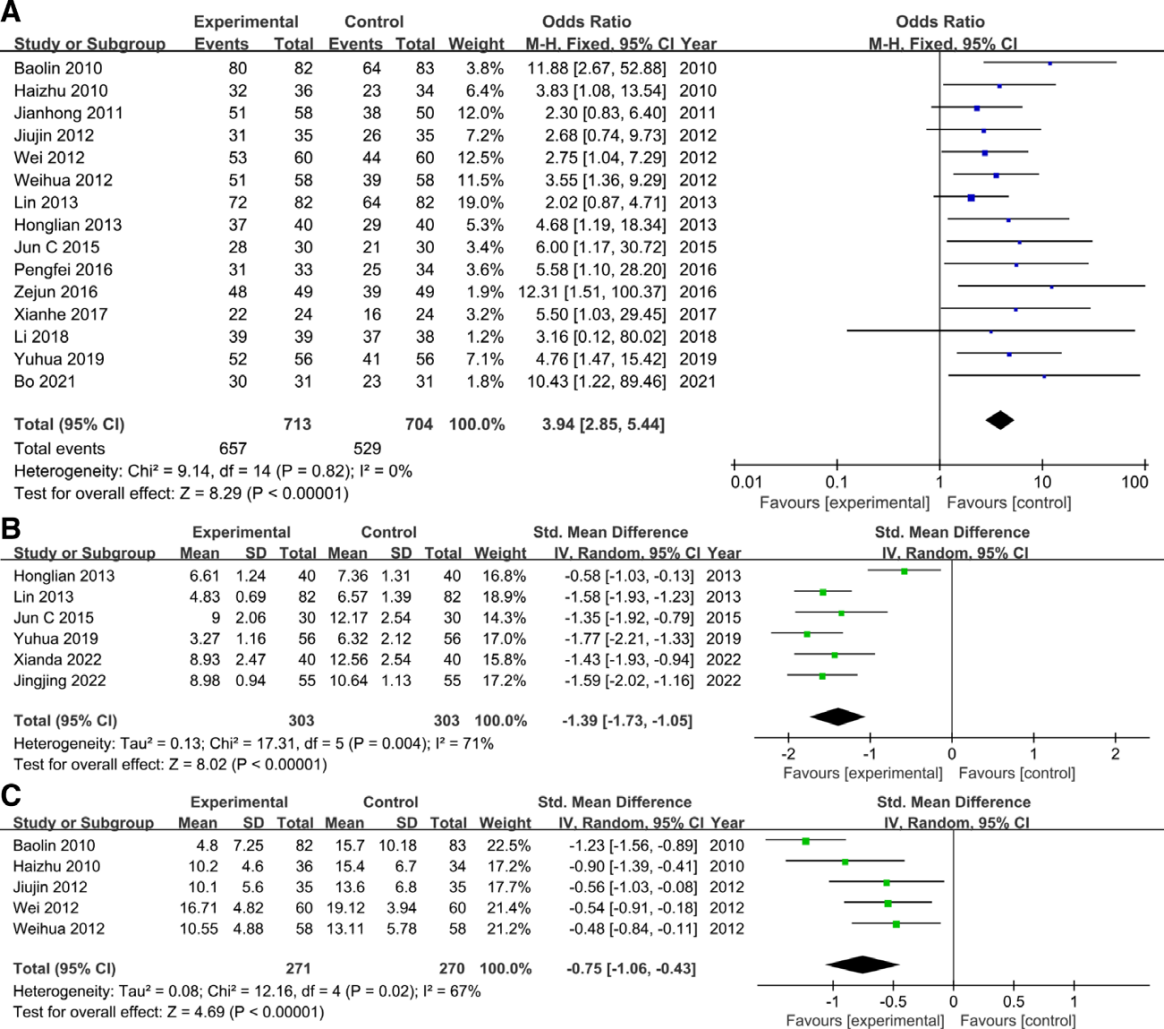
The forest plots assessing the impact of ERI plus SXN therapy versus ERI alone on the effectiveness, NIHSS, and NDS in patients with acute cerebral infarction. (A) Effectiveness. (B) NIHSS. (C) NDS. ERI = edaravone injection, NDS = neural function defect score, NIHSS = National Institute of Health Stroke Scale, SXN = Shuxuening injection.

A total of 15 RCTs^[32–46]^ evaluated and reported the efficacy after treatment. The heterogeneity test showed no significant heterogeneity among the included studies (*P* = .82, *I*^2^ = 0%). Therefore, a fixed-effects model was used for meta-analysis (Fig. 3A). The results showed that compared with the ERI single-agent group, the treatment effect of the combination group was improved, and the difference was statistically significant (odds ratio = 3.94, 95% CI: 2.85, 5.44; *P* < .00001).

#### 3.3.2. NIHSS.

Seven studies^[30,31,33,35,38–40]^ reported the prior and posttreatment NIHSS scores. The heterogeneity test (*P* = .004, *I*^2^ = 71%) indicated significant heterogeneity between studies, so a random-effects model was used for meta-analysis (Fig. 3B). The results showed that the difference was statistically significant (SMD = −1.39, 95% CI: −1.73, −1.05; *P* < .00001), suggesting that SXN combined with ERI has a better effect on reducing neurological damage than the control group.

#### 3.3.3. NDS.

Five studies^[41–43,45,46]^ reported changes in NDS after treatment. The heterogeneity test (*P* = .02, *I*^2^ = 67%) showed significant heterogeneity among studies, so a random-effects model was used for pooled analysis. Meta-analysis results (Fig. 3C) showed that the difference in NDS between the 2 groups was statistically significant (SMD = −0.75, 95%CI: −1.06, −0.43; *P* < .00001), indicating that the NDS improvement effect of the combination group was better than that of the control group.

#### 3.3.4. ADL.

Two studies^[34,39]^ reported changes in ADL after treatment. One reported the number of patients with improved ADL after treatment, and the other reported the level of ADL after treatment. Both studies showed that the improvement in ADL was better in patients of the combination group than in the control group (Supplemental Figure S1A, Supplemental Digital Content, http://links.lww.com/MD/I465).

#### 3.3.5. IL-6.

**Figure 4.**
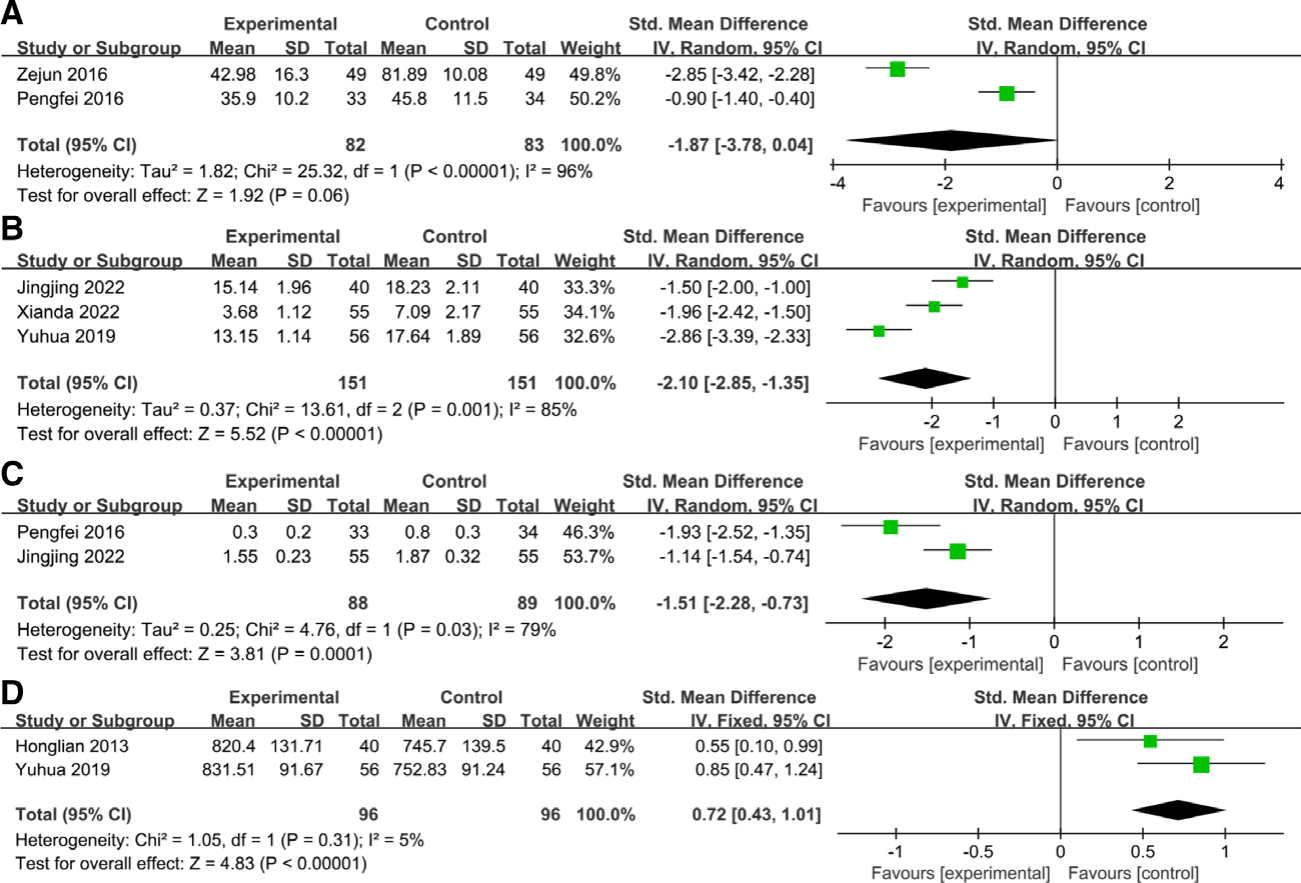
The forest plots assessing the impact of ERI plus SXN therapy versus ERI alone on the inflammatory markers and angiogenesis markers in patients with acute cerebral infarction. (A) IL-6. (B) NSE. (C) S100-beta. (D) VEGF. ERI = edaravone injection, NSE = neuron-specific enolase, SXN = Shuxuening injection, VEGF = vascular endothelial growth factor.

Two studies examined IL-6 expression levels after treatment.^[36,37]^ The heterogeneity test (*P* < .01, *I*^2^ = 96%) showed significant heterogeneity among studies. Therefore, a random-effects model was used for meta-analysis. The results showed that there was a difference in IL-6 levels between the 2 groups, but there was no statistical significance (SMD = −1.87, 95% CI: −3.78, 0.04; *P* = .06), suggesting that patients with SXN combined with ERI were more effective in reducing IL-6 levels than the control group (Fig. 4A).

#### 3.3.6. TNF-α.

One study examined posttreatment TNF-α levels.^[36]^ This study showed that in patients with acute cerebral infarction, the level of TNF-α in the combination group was significantly lower than that in the control group, suggesting that SXN combined with ERI had a better effect on reducing TNF-α than that of the control group.

#### 3.3.7. CRP.

One study presented changes in CRP levels before and after treatment.^[46]^ The results of this study showed that the difference in CRP levels between the 2 groups was statistically significant, suggesting that SXN combined with ERI was better than the control group in improving CRP (Supplemental Figure S1B, Supplemental Digital Content, http://links.lww.com/MD/I465).

#### 3.3.8. NSE.

Three studies assessed levels of NSE after treatment.^[30,31,33]^ The heterogeneity test (*P* = .001, *I*^2^ = 85%) indicated significant heterogeneity between studies. Therefore, a random-effects model was used for meta-analysis. The results showed that the difference was statistically significant (SMD = −2.10, 95% CI: −2.85, −1.35; *P* < .00001), suggesting that the combination group had a better effect on reducing NSE than the control group (Fig. 4B).

#### 3.3.9. S100-beta.

Two studies examined levels of S100-beta before and after treatment.^[31,37]^ Due to significant heterogeneity between studies (*P* = .03, *I*^2^ = 79%), a random-effects model was used for pooled analysis (Fig. 4C). Compared with the control group, the combination group had an advantage in reducing S100-beta, and the difference between the 2 groups was statistically significant (SMD = −1.51, 95% CI: −2.28, −0.73; *P* = .0001).

#### 3.3.10. VEGF.

Two studies assessed changes in VEGF levels after treatment.^[33,40]^ Since no significant heterogeneity was detected (*P* = .31, *I*^2^ = 5%), a fixed-effects model was used for analysis (Fig. 4D). The results showed that the VEGF level in the control group after treatment was higher than that in the combination group, and the difference was statistically significant (SMD = 0.72, 95% CI: 0.43, 1.01; *P* < .00001).

#### 3.3.11. Hemorheology.

##### 3.3.11.1. Whole blood high shear viscosity.

**Figure 5.**
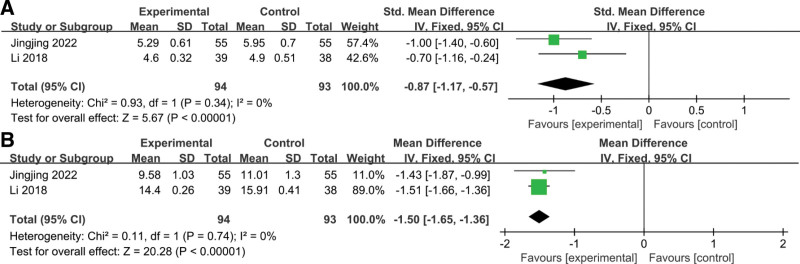
The forest plots of assessing the impact of ERI plus SXN therapy versus ERI alone on the hemorheology in patients with acute cerebral infarction. (A) Whole blood high shear viscosity. (B) Whole blood low shear viscosity. ERI = edaravone injection, SXN = Shuxuening injection.

Two studies reported changes in whole blood high-shear viscosity and whole blood low-shear viscosity after treatment, respectively.^[31,34]^ The heterogeneity test showed no significant heterogeneity between the study results (for whole blood high shear viscosity: *P* = .34, *I*^2^ = 0%; for whole blood low shear viscosity: *P* = .74, *I*^2^ = 0%). Therefore, fixed-effect models were used for analysis. The results showed that after treatment, whole blood high shear viscosity (SMD = −0.87, 95% CI: −1.17, −0.57; *P* < .00001) and whole blood low shear viscosity (SMD = −1.50, 95% CI: −1.65, −1.36; *P* < .00001) in the combination treatment group were significantly lower than those in the control group. These changes suggested that SXN combined with ERI was better than the control group in reducing blood viscosity in these patients (Fig. 5A and B).

### 3.4. Safety

Seven studies reported adverse events, but only 2 reported details.^[30,34,41–43,45,46]^ Descriptive analysis of adverse reactions was conducted in 5 studies, and the results of these studies showed that the types and incidence of adverse reactions were similar between the 2 groups.^[30,34,41–43,45,46]^ Descriptive analysis was used because of significant heterogeneity between studies. The study by Xianda et al^[30]^ found that the incidence of adverse reactions in the control group was significantly lower than that in the control group (*P* < .05). The study by Weihua et al^[41]^ showed that there was no significant difference in the incidence of adverse reactions between the 2 groups (*P* > .05).

### 3.5. Publication bias assessment

**Figure 6.**
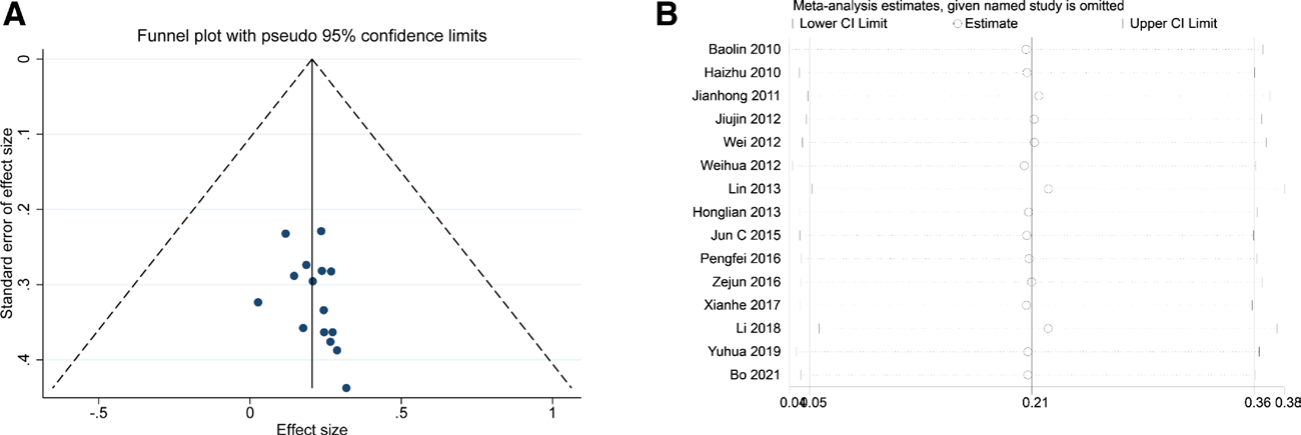
The results of (A) publication bias assessment and (B) sensitivity analysis.

The publication bias of the included literature was assessed by Egger

 test, Begg test, and funnel plot. The funnel plot of efficiency was symmetrical (Fig. 6A), and the *P* values of Egger and Begg tests were 0.09 and 0.17, respectively, indicating that significant publication bias was not found among the studies included in this analysis.

### 3.6. Sensitivity analysis

The robustness and reliability of meta-analysis outcomes were detected by sensitivity analysis. We used the analysis result of effectiveness and performed sensitivity analyses to assess whether the outcomes were stable after excluding 1 study once a time. The result (Fig. 6B) showed that the meta-analysis outcomes were not dramatically changed when compared to the overall estimate.

## 4. Discussion

Acute cerebral infarction is a common ischemic cerebrovascular disease in middle-aged and elderly people, which has the characteristics of high disability rate, high mortality rate, and low cure rate.^[1,20,32,47]^ Improving the blood circulation of brain tissue and protecting the function of brain cells is of great significance for the treatment of acute cerebral infarction.^[5,9,31,40]^ Edaravone, as a powerful free radical scavenger, improves blood circulation in brain tissue and delays disease progression.^[14,15]^ SXN is a Chinese patent medicine processed from Ginkgo biloba extract, which has the effect of promoting blood circulation.^[21,31]^ However, there is still a lack of strong evidence on the efficacy and safety of ERI combined with SXN in the treatment of acute cerebral infarction. The results of this study show that ERI combined with SXN can improve the curative effect, improve brain nerve function damage and hemorheology, and reduce the expression level of inflammatory factors. Meanwhile, the combination group did not significantly increase the incidence of adverse reactions and showed acceptable safety.

Due to the serious condition of acute cerebral infarction, drug combination therapy is currently used in clinical practice to improve the clinical effect. Among them, traditional Chinese medicine treatment can be combined with other treatments for this disease.^[26,29,48]^ It can not only relieve clinical symptoms in a short time, but at the same time, the active ingredients in traditional Chinese medicine are beneficial to the improvement of blood circulation.^[26,29,48]^ The incidence of neurological impairment such as disability and language impairment can also be improved.^[1,49]^ The results of this study showed that after SXN plus ERI treatment, the treatment efficacy, NIHSS score, NDS, ADL, S100-beta protein, NSE, and other nerve injury markers were improved in patients with acute cerebral infarction when compared to those of the ERI group. This study further illustrates that based on ERI, the addition of SXN can improve the degree of neurological damage and protect nerve function in patients with acute cerebral infarction.

Patients with cerebral infarction generally have abnormal inflammatory factors and hemorheological indexes, which are mainly manifested as increased levels of inflammatory factors and significantly increased whole blood viscosity and other indexes.^[48–50]^ In an inflammatory environment, changes such as increased viscosity and slow flow rate will further aggravate neuronal cell death and brain tissue hypoxic-ischemic damage.^[29–31]^ Therefore, actively improving hemorheology is important for treatment. The results of this study showed that hemorheological indexes such as whole blood high shear viscosity and whole blood low shear viscosity were decreased in the 2 groups after treatment, and the above indexes in the observation group were lower than those in the control group. These may have positive effects on the improvement of the patients’ prognosis and quality of life.

This study also has some limitations. First, although the included studies are all RCTs, the sample size of each study is relatively small and characterized by single-center. Allocation concealment and blinding were not mentioned in the included studies. Such limitation may be a major source of bias in meta-analyses. Second, the clinical baseline characteristics of the included studies, such as lifestyle, age, and gender, may contribute to an increased risk of heterogeneity. Third, the outcome indicators of some studies did not present specific values, and these may increase the bias risk of selective reporting. Nevertheless, we provide clinical evidence for SXN combined with ERI in the treatment of acute cerebral infarction.

In conclusion, SXN combined with ERI can improve the levels of nerve injury markers, inflammatory factors, and hemorheology indexes in patients with acute cerebral infarction, resulting in better nerve function and therapeutic effects. However, large-sample, multi-center studies are still needed for further verification in the future.

## Author contributions

**Conceptualization:** Liang-Da Li.

**Data curation:** Liang-Da Li, Yue Zhou.

**Formal analysis:** Liang-Da Li, Yue Zhou, Shan-Fen Shi.

**Investigation:** Liang-Da Li.

**Methodology:** Liang-Da Li, Shan-Fen Shi.

**Project administration:** Liang-Da Li.

**Software:** Liang-Da Li, Shan-Fen Shi.

**Supervision:** Liang-Da Li.

**Validation:** Liang-Da Li, Yue Zhou.

**Visualization:** Liang-Da Li, Yue Zhou.

**Writing – original draft:** Liang-Da Li, Yue Zhou, Shan-Fen Shi.

**Writing – review & editing:** Liang-Da Li.

## Supplementary Material


