# Supplementary material for: Edaravone combined with Shuxuening versus edaravone alone in the treatment of acute cerebral infarction: A systematic review and meta-analysis
Source: Medicine (Baltimore). 2023 Mar 3;102(9):e32929. doi: 10.1097/MD.0000000000032929 (PMC9981379; doi:10.1097/MD.0000000000032929)

Supplemental Figure 1 The forest plots of assessing the impact of ERI plus SXN therapy versus ERI alone on the ADL (A) and CRP (B) in patients with acute cerebral infarction.

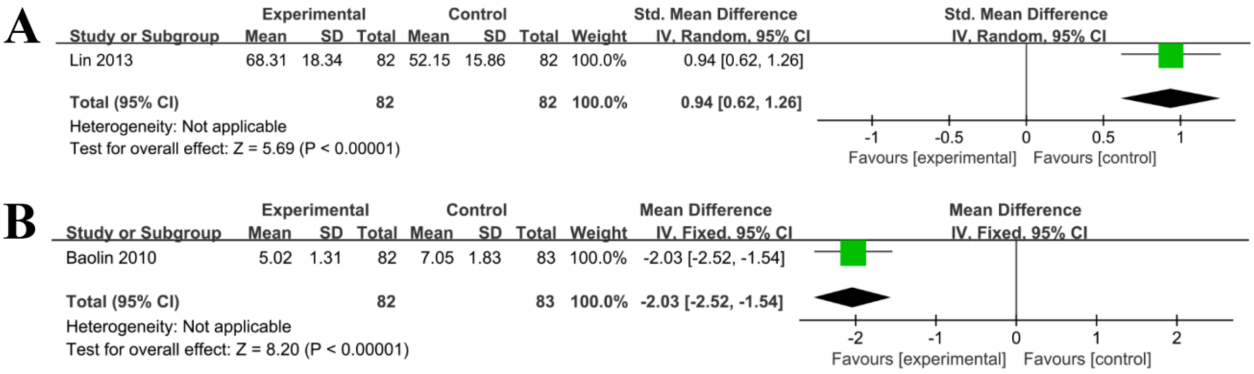

Supplement: Supplementary file 1 [file medi-102-e32929-s001.pdf]
